# Measuring the Impact of Limb Asymmetry on Movement Irregularity and Complexity Changes During an Incremental Step Test in Para-Swimmers Using Inertial Measurement Units

**DOI:** 10.3390/s25113297

**Published:** 2025-05-24

**Authors:** Matthew Slopecki, Julien Clément, Mathieu Charbonneau, Julie N. Côté

**Affiliations:** 1 Department of Kinesiology and Physical Education, McGill University, Montreal, QC H2W 1S4, Canada; julien.clement@etsmtl.ca (J.C.); julie.cote2@mcgill.ca (J.N.C.); 2 Institut National du Sport, Montreal, QC H1V 3N7, Canada; mcharbonneau@insquebec.org; 3 Département de Génie des Systèmes, École de Technologie Supérieure, Montréal, QC H3C 1K3, Canada

**Keywords:** swimming, anthropometry, IMU, fatigue, variability, movement complexity, movement irregularity, wearable sensors, performance analysis, para-swimming

## Abstract

Wearable technology can nowadays be used to improve para-swimming coaching; however, the extent to which individual anatomy affects features of swimming variability is unclear. Six paralympic swimmers were recruited, their upper-limb segment lengths were measured, and their absolute bilateral limb asymmetry indices (${AbsLAI}_{UL}$) were calculated. They were instrumented with a sacrum-worn inertial measurement unit and performed an in-water, fatiguing, freestyle aerobic test at incrementally faster paces. Stroke-to-stroke outcome and execution variability were calculated, respectively, using sample entropy (SampEn) and fractal dimension (FD) on forward and mediolateral linear acceleration signals. Significantly increased perceived exertion scores (F(4,28) = 154.1, *p* < 0.001) were observed. Execution and outcome variability increased in the forward (SampEn = F(4,25) = 11.86, *p* < 0.001; FD = F(4,24) = 6.17, *p* = 0.001) and mediolateral (SampEn = F(4,25) = 9.46, *p* < 0.001; FD = F(4,24) = 27.64, *p* < 0.001) directions. Modelling of FD (only) improved with ${AbsLAI}_{UL}$ as a covariate (forward = F(1,24) = 9.68, *p* = 0.005; mediolateral = F(1,24) = 8.57, *p* = 0.021), suggesting that ${AbsLAI}_{UL}$ affects only execution, but not outcome, variability. This information could help coaches determine which coordination indices should be personalized when monitoring variability during para-swimming training.

## 1. Introduction

Freestyle swimming is a bilateral, cyclical movement task that requires coordinated movement patterns of the upper limbs to maximize forward propulsion while minimizing movement in other planes. Para-swimmers are a unique population who may have physical and/or neurological impairments that impact the symmetry of their bilateral anatomy, in turn impacting their ability to create symmetrical propulsive movement patterns. As such, those para-swimmers must develop motor control strategies that account for any structural or functional impairment in their ability to produce symmetrical movement patterns in order to optimize their effort towards forward movement.

Some of the control strategies developed to improve swimming efficiency can target motor variability, defined as the natural variation that occurs across repetitions of a movement task [1]. In some areas of the literature, motor variability has also been considered as a motor control strategy to mitigate the challenges induced by performing fatiguing tasks during training, whereby task-specific movement adaptations are produced to help optimize a task-related performance goal while managing the exercise-induced fatiguing effects on joint and muscles [2]. In other words, variability of the execution of a task is used to stabilize the outcome variability of a movement [3]. In the context of swimming, this would refer to how variabilities of limb movement patterns are used to stabilize propulsive patterns at the centre of mass (CoM) of swimmers.

Several analytical approaches can be applied to movement (kinematic) signals to infer changes in execution and outcome variability. Two such methods are sample entropy [4], quantifying the inter-cycle irregularity of cyclical movement, and fractal dimension [5], quantifying the amount of complexity present in a signal. In swimming, SampEn measured near the CoM can quantify the repeatability or irregularity of propulsive patterns [6,7], or outcome variability [3]. Additionally, FD measured near the CoM can provide information on the complexity of a swimmer’s motor behaviour [7,8], which may reflect the magnitude of flexible adaptations of coordinated limb movements, or execution variability [3]. While both irregularity and complexity of acceleration patterns at the CoM are nonlinear features of variability, movement irregularity quantifies the likelihood that the next propulsion would be the same as the current one, while fractal dimension quantifies richness of temporal structures at different time scales [9], or the adaptivity of movement patterns. As such, the outcome variability in the current study reflects the repeatability of the swimming pattern, while the execution variability represents the adaptive changes in swimming patterns. Viewed through the theory of motor abundance [10], increased execution variability while outcome variability remains constant would suggest it is beneficial to task performance, while increased execution variability with increased outcome variability (irregularity) would suggest that these adaptive changes had negative impacts on the task.

Based on the previous literature from other fields, effective fatigue mitigation strategies would then draw from changes in FD in ways that would not negatively affect (increase) SampEn in order for performance to be maintained despite fatigue being gradually developed when performing a repetitive task. Indeed, previous studies of other cyclical tasks performed by able-bodied participants showed that fatigue induced through running led to increased mediolateral range of motion of the centre of mass [11] and increased motor variability that negatively impacted task performance, attributed to a decrease in control of the centre of mass [12]. This highlights the potential use of studying control of the centre of mass in complex, multi-segment movement patterns as a surrogate measure of outcome variability. Previous research has also applied these entropy and fractal dimension analyses to swimming to determine differences in expertise level and swimming stroke styles [7,13]. However, they used cable displacement-based techniques with a belt attached to the swimmers’ hips to measure those features, which may have restricted the swimmers’ trunk motion and could have affected the ecological validity of their measurements.

Recently, wearable technologies, such as inertial measurement units (IMU), have become a popular tool for swimming analyses [14,15,16,17,18]. Compared to previous swimming motion analysis techniques, they provide tridimensional acceleration data that can be used to quickly compute various movement metrics. In turn, these metrics can inform coaches and athletes on the quality of swimming movements [19,20,21]. Previous studies using a single sacrum-worn IMU to analyse swimming have been able to estimate instantaneous velocity [22,23] and swim stroke mechanics (such as instantaneous stroke rate, distance per stroke, and upper-limb movement timing symmetry) [18,24,25] in able-bodied samples. Additionally, estimation of instantaneous velocity [17] and multiple swim stroke mechanics [16] have also been validated in para-swimmers. Furthermore, a single sacrum-worn IMU was sufficient in order to obtain individualized training-based, performance-relevant values for para-swimmers [26]. Few studies have used a single sacrum-worn IMU to analyse swimming-related variability metrics in able-bodied swimmers [19,20], but no research to date has investigated how features of variability evolve during the performance of fatiguing tasks in paralympic swimmers. Finally, no research has investigated if individual features of anthropometric asymmetry may affect the various components of swimming-related motor variability.

The aim of the present study was to use wearable inertial sensor data to investigate how anthropometric limb asymmetry in para-swimmers affects the motor variability characteristics of movement irregularity and complexity of CoM acceleration responses to performing a fatiguing task. We hypothesized that movement outcome variability characteristics would become more irregular (i.e., entropy would increase) and movement execution variability characteristics would become more complex (i.e., fractal dimension would increase) as fatigue gradually develops. We further hypothesized that accounting for individual limb asymmetry scores would improve the statistical modelling of responses in movement irregularity and complexity outcomes.

## 2. Materials and Methods

### 2.1. Participants

Six elite (sex = 3m, 3f; age = 22.5 ± 3.2 years; height = 155.1 ± 29.4 cm; body mass 60.2 ± 19.4 kg) paralympic swimmers were recruited to participate in this study. All swimmers were classified by the World Para Swimming Classification Panels and competed at an international level. Details of the impairment and swimming classification groups are reported in Table 1. Informed consent was obtained from all participants. This study was conducted according to the guidelines of the Declaration of Helsinki and approved by the Institutional Review Boards of McGill University (protocol code 22-05-021) and École de Technologie Supérieure (protocol code H20221001).

### 2.2. Instrumentation

A single IMU (±16 g, 120 Hz, Xsens Dot, Xsens Technologies, Enschede, The Netherlands) was placed between the participant’s posterior superior iliac spines. A Tegaderm adhesive patch (3M Health Care, Minneapolis MN, USA) was used to secure the IMU to the participant’s skin, ensuring firm attachment and minimizing sensor movement during data collection. The IMU had dimensions of 36 × 30 × 11 mm and a mass of 3.15 g. The IMU measured tri-axial linear accelerations. Data were recorded offline on the IMU onboard memory. To ensure adequate statistical power with the current analyses, only acceleration signals in the cranio-caudal (referred to as “forward” in the present study) and mediolateral directions were analysed in the present study.

### 2.3. Protocol

Bilateral measurements of upper-limb segment lengths were taken. Three manual measurements were taken of left and right (1) upper arm (acromioclavicular joint to the lateral epicondyle of the elbow), (2) forearm (lateral epicondyle of the elbow to the mid-point between the medial and lateral styloid processes), and (3) hand (mid-point between the medial and lateral styloid processes to the most distal point of the middle finger) lengths by a trained operator. For swimmers with undeveloped or amputated limbs, measurements were taken to the most distal part of the segment.

The participants performed a freestyle aerobic step test, as described by Anderson [27]. Briefly, the step test comprised 5 incrementally faster repetitions of freestyle swimming, referred to as steps, over 200 m (*n* = 4), 150 m (*n* = 1) or 100 m (*n* = 1), based on the swimmer’s impairment. The target times for each of the 5 steps were based on the swimmer’s personal best time for the given distance. The target time of the final step was defined as the swimmer’s personal best time rounded up to the nearest minute. The target time for the first step was 20 s slower than the final step, and each of the steps between were swum at a target time of 5 s faster than the previous step [27]. Thus, the test aimed to assess how an athlete is able to execute the task at progressively faster speeds in a state where the incremental steps gradually induced a fatiguing effect on the muscles. In the present study, the collected measures reflect both changes in fatigue and pace in a way that does not permit distinguishing between them. All tests were completed in an Olympic-size (long course; 50 m) swimming pool, which was each swimmer’s usual training environment. Ratings of perceived exertion (RPE) were also measured after each step of the test using the Borg CR-10 scale [28].

### 2.4. Data Processing

#### 2.4.1. Anthropometric Data

Mean (±SD) averages of each limb segment length measurement were calculated. Left and right upper-limb lengths were calculated as the sum of the mean upper arm, forearm, and hand segment lengths for the left and right sides. The Limb Asymmetry Index (LAI) [29] between the left and right upper-limb lengths (${LAI}_{UL}$) were calculated using Equation (1). Positive ${LAI}_{UL}$ values represent a greater left upper-limb length in proportion to their right length.

(1) $${LAI}_{UL}=\frac{\left(Left\;Limb\;Length-Right\;Limb\;Length\;\right)}{\left(Left\;Limb\;Length+Right\;Limb\;Length\right)}\times 100$$

Finally, absolute values of ${LAI}_{UL}$ were calculated (${AbsLAI}_{UL}$), representing a non-directional magnitude of asymmetry.

#### 2.4.2. Swimming Data

All swimming data processing were completed in MATLAB (Version 2023a, Mathworks, Natick, MA, USA). Raw linear acceleration signals from the x and y axes defined the forward (cranio-caudal) and medio-lateral (ML) data. To ensure that the analyses focused on steady-state swimming, starts, flip turns, and the first and last two stroke cycles of each lap were manually removed from the data. The remaining cyclical swimming data were vertically concatenated using MATLAB’s “vertcat” function. A sample entropy (SampEn) analysis [4] was applied to the subsequent raw forward and mediolateral linear acceleration signals to quantify movement irregularity. N was defined as the length of the dataset; embedding dimension (m) was defined as 2 using the false nearest neighbours method [30]; and the tolerance limit, r, was set as 0.1 × SD. SampEn ranges from 0 (perfectly predictable) to infinity (completely unpredictable). A fractal dimension (FD) analysis was applied to the raw forward and mediolateral linear acceleration signals, using the Higuchi algorithm [5]. FD ranges from 1 (low complexity in structure, such as a straight line) to 2 (highly complex patterns). In freestyle swimming, expert able-bodied swimmers had approximate entropy, analogous to SampEn, and FD values of 0.66 ± 0.12 and 1.84 ± 0.08, respectively [13]. Percentage of baseline SampEn and FD values were calculated, where the baseline was defined as step 1 values. This quantified the relative changes in irregularity and complexity, defined as SampEn% and FD%.

### 2.5. Statistical Analyses

Statistical analyses were completed using R [31]. An ANOVA model compared step (1:5) on RPE values, using base R’s “aov” function. ANOVA models also compared the main effects of step (1:5) with and without the covariate ${AbsLAI}_{UL}$ on SampEn% and FD% using forward and mediolateral acceleration data. Post hoc Tukey’s HSD was run on significant main effects, using base R’s “TukeyHSD” function. Finally, given that the variability results were analysed relative to step 1 data, to account for potential inter-individual differences in non-fatigued swimming variability as a function of LAI, we ran Spearman’s rank correlations between ${AbsLAI}_{UL}$ and the step 1 values of SampEn and FD. Benjamini–Hochberg corrections were applied to critical alpha values to account for multiple comparisons [32].

## 3. Results

### 3.1. Rate of Perceived Exertion (RPE)

**Figure 1:**
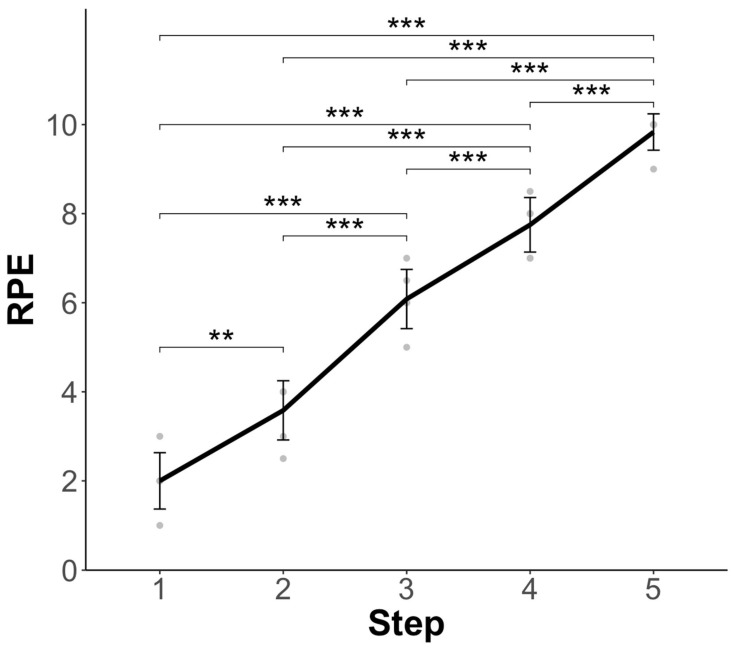
Mean ± SD ratings of perceived exertion (RPE) values at each step of the step test. The grey circles show all RPE values. Post hoc differences in RPE by step for each participant, after a significant main effect of step in the analysis of variance (ANOVA) model. Significant post hoc differences are displayed with significance brackets. *** denotes statistical significance at *p* < 0.001, ** denotes statistical significance at *p* < 0.01.

RPE increased significantly by step (F(4,28) = 154.1, *p* < 0.001), with significant differences between all steps (*p* < 0.05). Mean (±SD) RPE values with post hoc differences are shown in Figure 1.

### 3.2. Limb Asymmetry

The average measurements of left and right arm lengths, and ${AbsLAI}_{UL}$ values are displayed in Table 2.

### 3.3. Motor Variability

#### 3.3.1. Baseline (Step 1) Values

**Figure 2:**
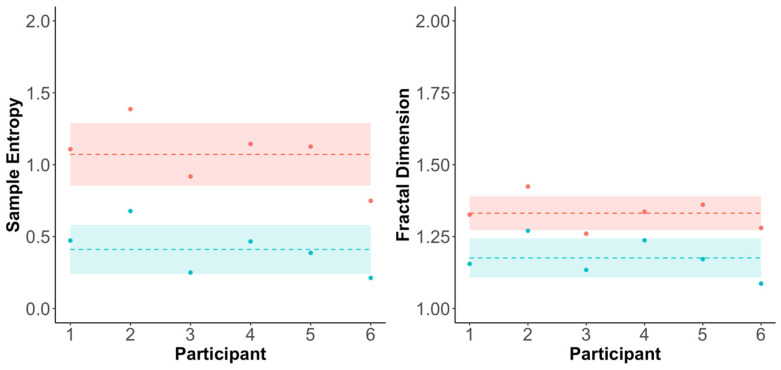
Baseline values for forward (red circles) and mediolateral (blue circles) components of SampEn and FD. Mean (±SD) values shown as dashed line (±cloud).

The baseline values for SampEn and FD are shown in Figure 2. The mean (±SD) baseline SampEn values were 1.07 (±0.22) and 0.41 (±0.17) in the forward and mediolateral directions, respectively. The mean (±SD) baseline FD values were 1.33 (±0.06) and 1.18 (±0.07) in the forward and mediolateral directions, respectively.

#### 3.3.2. Sample Entropy (SampEn)

**Figure 3:**
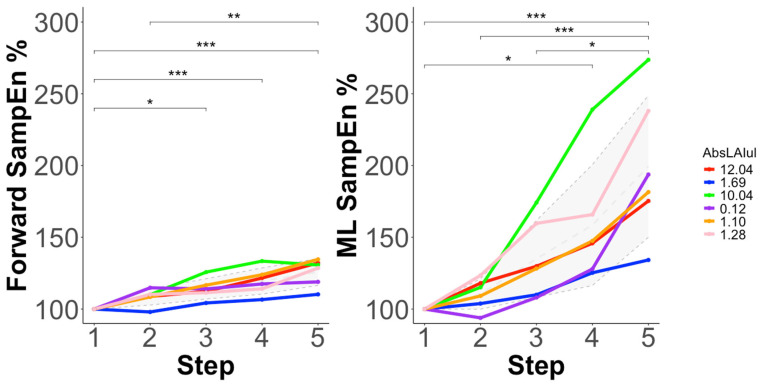
Post hoc differences in relative change in sample entropy (SampEn%) by step (1–5) for each participant, after a significant main effect of step in the analysis of variance (ANOVA) models. The mean (±SD) response is shown with a grey dashed line (SD = grey cloud). Significant post hoc differences are displayed with significance brackets. *** denotes statistical significance at *p* < 0.001, ** denotes statistical significance at *p* < 0.01, and * denotes statistical significance at *p* < 0.05.

SampEn% ANOVA models with and without ${AbsLAI}_{UL}$ as a covariate are displayed in Table 3. Significant main effects of step were observed in all models. ${AbsLAI}_{UL}$ was not a significant covariate in the forward or mediolateral SampEn% ANOVA models. The post hoc changes are displayed in Figure 3. No correlations (forward = r(4) = −0.31, *p* = 0.56; ML = r(4) = −0.20, *p* = 0.71) were observed between the step 1 SampEn values and ${AbsLAI}_{UL}$.

#### 3.3.3. Fractal Dimension (FD)

**Figure 4:**
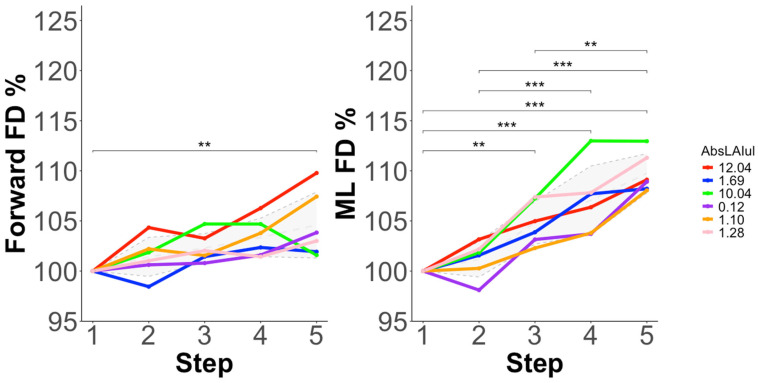
Post hoc differences in relative change in fractal dimension (FD%) by step (1–5) for each participant, after a significant main effect of step in the analysis of variance (ANOVA) models. The mean (±SD) response is shown with a grey dashed line (SD = grey cloud). Significant post hoc differences are displayed with significance brackets. *** denotes statistical significance at *p* < 0.001, ** denotes statistical significance at *p* < 0.01.

FD% ANOVA models with and without ${AbsLAI}_{UL}$ as a covariate are displayed in Table 4. Significant main effects of step were observed in all models. ${AbsLAI}_{UL}$ was a significant covariate in both the forward and mediolateral FD% ANOVA models. The post hoc changes are displayed in Figure 4. No correlations (forward = r(4) = −0.37, *p* = 0.50; ML = r(4) = −0.31, *p* = 0.56) were observed between the step 1 FD values and ${AbsLAI}_{UL}$.

## 4. Discussion

In this study, we investigated how anthropometric upper-limb asymmetries contributed to changes in movement irregularity (SampEn) and complexity (FD) from start to finish of an incremental, fatiguing step test. The results show that the baseline values for movement irregularity (outcome variability) and complexity (execution variability) were higher in the forward direction, compared to in the mediolateral direction. Additionally, both movement irregularity and complexity of propulsive patterns increased with step, in both the forward and mediolateral directions. Anthropometric asymmetries contributed to relative changes in movement complexity (execution variabilities), but not movement irregularity (outcome variabilities). Finally, no correlation was observed between baseline variability values and limb asymmetry.

At baseline (step 1), movement irregularity and complexity were higher in the forward direction, compared to the mediolateral direction. This may be a result of the movement task goal, whereby swimmers move predominantly in the forward direction. Additionally, the effects of drag may result in inherently higher irregularity of the trunk during forward swimming, due to the constant balance between positive and negative forward propulsion. Finally, no correlations were observed between the magnitude of limb asymmetry and levels of baseline movement irregularity and complexity. This may be the result of motor control strategies that are effectively developed by these experienced athletes and that account for any anthropometric differences between-limb when unfatigued. In previous studies, mediolateral movement irregularity of stride parameters, quantified by SampEn, during regular walking were significantly higher in lower-limb amputees compared to controls [33]. Furthermore, stability components in both the forward and mediolateral directions were significantly lower in amputees, suggesting greater flexibility in movement execution patterns [33]. However, the authors did not report whether inter-individual differences were present among the amputees. In our study, the fact that no component of baseline motor variability was impacted by the amount of upper limb bilateral asymmetry is interesting and suggests that for non-fatigued swimming, this metric could be used to monitor all para-swimmers in the present sample equally. However, this hypothesis should be confirmed through future studies that have access to a homogenous sample of para-swimmers with physical impairments affecting between-limb asymmetry.

However, the same cannot be said as the incremental step test progressed, which does show an impact of limb asymmetry, although not for all the tested measures. To start, our results do show that the different features of variability seem to evolve differently as the incremental step test progresses. Indeed, increases in outcome (irregularity) variability patterns, as quantified by SampEn, were observed in both the forward and mediolateral movement directions. In fact, movement irregularity (outcome variability) had greater relative increases from step one values than movement complexity (execution variability) in both directions. Additionally, relative increases in movement irregularity were greater in the mediolateral direction compared to the forward direction (Figure 3). While running involves different mechanical patterns to swimming, it represents another sport that is based on continuous cyclical, alternating limb movement patterns that are produced in order to propel the body forward, which could help to ground the current study’s findings. For example, runners increased their trunk’s motor variability patterns after completing a fatiguing protocol, suggesting that fatigue negatively affected the control of their centre of mass [12]. Moreover, in a similar study, the authors found that this was particularly the case for mediolateral centre of mass movement increases with fatigue [11]. This supports the interpretation of our findings in suggesting that control of the CoM, especially in the mediolateral direction, could be a good strategy to help mitigate the development of fatigue induced in forward-movement cyclical sports, such as running and swimming, and that for better performance, its outcome measure of irregularity (SampEn) could be targeted and further trained as part of fatigue mitigation strategies. One such approach may be a targeted intervention developing core stability and strength endurance of shoulder musculature in this population, but future studies are needed to determine which coaching-specific interventions can be developed in order to target the irregularity (outcome variability) changes observed in the current study.

Our results also show that movement complexity (execution variability) increased in both the forward and mediolateral directions as the incremental test progressed, with more distinct between-step changes in the mediolateral direction (Figure 4). This may be the result of increased execution variability, or adaptive swimming patterns. Following the motor abundancy hypothesis [10], increased variability of individual limb movement patterns may have been used as a motor control strategy to mitigate the negative effects of ongoing fatigue on continuous forward movement, the performance variable in swimming. However, our results suggest that the strategy of modifying movement irregularity as the incremental test developed may have its limits as a fatigue mitigation strategy. In particular, we observe that increases in execution variability (complexity) in the forward direction seemed to plateau well before the final step for the majority of participants, with no significant differences observed between steps 3 and 5 (Figure 4). This reinforces the interpretation that other fatigue mitigation strategies may have been developed starting from that point on, possibly to avoid further negatively impacting outcome variability features that would in turn negatively further affect performance.

At this point, the described strategies are discussed across the group; however, some of our results do show an impact of individual asymmetry scores on changes in variability. Indeed, taking into account the magnitude of asymmetry between upper-limb lengths did have an impact on some results: including ${AbsLAI}_{UL}$ as a covariate did improve modelling of changes in movement complexity (execution variability), but not movement irregularity (outcome variability), in both the forward and mediolateral directions. Fractal dimension, the measure of complexity, quantifies the amount of execution variability or adaptive variability in individual limb movement patterns. In the present study, participants with larger upper-limb asymmetries were those with Dysmeliac upper-limb and hand segments. Therefore, it is possible that reduced degrees of freedom at the upper limb were accounted for by increased motor variability strategies at the level of execution variability, or individual limb movements. However, accounting for limb asymmetry indices did not improve our statistical models of forward and mediolateral movement irregularity, or outcome variability. In other words, even if strategies of execution vary according to individual limb asymmetry, the end result does not. This further reinforces that individuals may choose from a range of potential motor solutions in planning to execute a performance, and this may be especially true for elite athletes with highly unique features such as para-swimmers. Therefore, general outcome features that affect drag, such as irregularity (outcome variability) of the centre of mass movement during swimming, may be considered as a group-based intervention metric, whereas complexity-based (execution variability) variables such as fractal dimension, should be used in a personalized way, according to each swimmer’s unique characteristics. The good news is that all those measures can be extracted rapidly from one sacrum-worn sensor, with adequate training to help the sport scientist make efficient choices in their coaching intervention practice.

## 5. Limitations and Future Work

We are aware of a number of limitations that could have impacted the results of the present study. Firstly, the sample size is low (*n* = 6). However, the sample represents the entire population of para-swimming athletes that were training with the studied group. Additionally, the sample is heterogenous in their types of impairments. As such, a high level of inter-subject variability exists in the dataset, although this too is representative of the studied population. To mitigate heterogeneity and attempt to extract general, group-based observations, we modelled the changes in motor variability outcomes relative to each individual’s values in the first step of the step test. However, other differences in type of impairments may also introduce error into our modelling of limb asymmetry on outcome variability (irregularity), where anthropometry may not explain all of the underlying causes for subject-specific differences in motor variability adaptations during incremental fatiguing tests. In the current study, only two of the six participants had limb asymmetry values considerably larger than the typical range, although the current literature on this topic is limited [34,35]. Additionally, the magnitude of ${AbsLAI}_{UL}$ values were clustered into high (*n* = 2) and low (*n* = 4) groups. As such, hypotheses about the magnitude of ${AbsLAI}_{UL}$ must be confirmed by future research studying a more homogenous population of para-swimmers such as those with Dysmeliac upper limbs. Furthermore, it must be acknowledged that during the performance of the chosen incremental step test, pace-related changes may also contribute to the observed changes in variability, to an extent that cannot be distinguished from any muscle fatigue effects. Due to data limitations, it was not possible in the current study to control for changes in swimming velocity. Nevertheless, we believe that our data can have practical use, in reflecting what happens during the performance of a test regularly completed by elite para swimmers. Future work should determine the effects of fatigue, using a similar protocol, with athletes swimming a consistent velocity between steps, as a way to tease out the effects of fluctuations in speed from those of muscle fatigue. Lastly, we used a single IMU placed on the sacrum of the para-swimmers to infer changes in individual limb movements (via complexity measures), but we did not specifically measure the changes in these limbs throughout the test. As such, some errors may have been introduced into our interpretations of execution variabilities. Future research should confirm this by directly measuring elemental and performance variabilities in this context, as well as evaluating the impact on IMU size and positioning on movement outcomes.

## 6. Conclusions

Our study further reinforces previous work in showing that it is possible to quantify motor variability responses using inertial technologies. Through this methodology, we have determined, for the first time, that anthropometric upper-limb symmetries contribute to increases in forward and mediolateral movement complexity, or execution variability, in paralympic swimmers completing an incremental step test. This implies that subject-specific anthropometric asymmetries will affect how para-swimmers can flexibly adjust movement patterns to mitigate perturbations to performance. However, the results of the current study may be influenced by differing paces at each step of the incremental protocol and from heterogeneity of the sampled para-swimmers. As such, future studies with statistically robust designs should directly test if larger magnitudes of asymmetry result in an earlier onset of the negative effects of fatigue, in protocols that isolate fatigue from swimming velocity. Coaches may want to consider the magnitude of limb asymmetries when programming training plans for para-swimmers to accommodate for individual differences in motor variability responses to delay fatigue-related performance reductions.

## Figures and Tables

**Table 1:** Details of impairments and para-swimming classification levels.

Swimmer ID	Impairment	Class
1	Right femoral–fibula–ulnar syndrome; Dysmeliac right upper limb	S8
2	Intellectual Impairment	S14
3	Dysmelia, congenital left-hand amputee	S10
4	Cerebral palsy	S9
5	Pseudoachondroplasia	S5
6	Achondroplasia dwarfism	S6

Note: For freestyle swimming, the World Para Swimming classifications for physical impairment range from S1 to S10. S14 refers to an intellectual impairment. A lower number indicates a more severe activity limitation than a higher number.

**Table 2:** Limb asymmetry index results for each swimmer.

Swimmer ID	Left Arm Length (cm)	Right Arm Length (cm)	$Abs{LAI}_{UL}$
1	79.90	62.73	12.04
2	88.10	91.13	1.69
3	66.43	81.27	10.04
4	80.17	80.37	0.12
5	45.77	44.77	1.10
6	51.40	50.00	1.28

**Table 3:** Analysis of variance (ANOVA) model results for main effects of step (1–5), with and without Absolute Limb Asymmetry (${AbsLAI}_{UL}$) as a covariate on percent change in sample entropy (SampEn%). F-score, degrees of freedom, and significance (*p*) are presented for the forward and mediolateral (ML) directions.

Direction	Covariate	Step	${AbsLAI}_{UL}$
Forward	With	F(4,24) = 13.11, *p* < 0.001 ***	F(1,24) = 3.64, *p* = 0.07
	Without	F(4,25) = 11.86, *p* < 0.001 ***	
ML	With	F(4,24) = 10.28, *p* < 0.001 ***	F(1,24) = 3.16, *p* = 0.09
	Without	F(4,25) = 9.46, *p* < 0.001 ***	

Note: *** denotes statistical significance at *p* < 0.001 for ANOVA model main effects.

**Table 4:** Analysis of variance (ANOVA) model results for main effects of step (1–5), with and without Absolute Limb Asymmetry (${AbsLAI}_{UL}$) as a covariate on relative changes in fractal dimension (FD%). F-score, degrees of freedom, and significance (*p*) are presented for forward and mediolateral (ML) directions.

Direction	Covariate	Step	${AbsLAI}_{UL}$
Forward	With	F(4,24) = 6.17, *p* = 0.001 **	F(1,24) = 9.68, *p* = 0.005 **
	Without	F(4,25) = 4.58, *p* = 0.009 **	
ML	With	F(4,24) = 27.64, *p* < 0.001 ***	F(1,24) = 8.57, *p* = 0.021 *
	Without	F(4,25) = 21.21, *p* < 0.001 ***	

Note: *** denotes statistical significance at *p* < 0.001, ** denotes statistical significance at *p* < 0.01 and * denotes statistical significance at *p* < 0.05 for ANOVA model main effects.

## Data Availability

The data presented in this study are available from the corresponding author on request. The data are not publicly available due to confidentiality of the sampled participants.
